# Deep Learning Algorithm-Based MRI Image in the Diagnosis of Diabetic Macular Edema

**DOI:** 10.1155/2022/1035619

**Published:** 2022-03-04

**Authors:** Xiuping Han, Juan Tan, Yumei He

**Affiliations:** ^1^Department of General Medicine, Affiliated Hospital of Yan'an University, Yan'an 716000, Shaanxi, China; ^2^Department of Traditional Chinese Medicine, Affiliated Hospital of Yan'an University, Yan'an 716000, Shaanxi, China

## Abstract

This study investigates the value of magnetic resonance imaging (MRI) based on a deep learning algorithm in the diagnosis of diabetic macular edema (DME) patients. A total of 96 patients with DME were randomly divided into the experimental group (*N*  = 48) and the control group (*N*  = 48). A deep learning 3D convolutional neural network (3D-CNN) algorithm for MRI images of patients with DME was designed. The application value of this algorithm was comprehensively evaluated by MRI image segmentation Dice value, sensitivity, specificity, and other indicators and diagnostic accuracy. The results showed that the quality of MRI images processed by the 3D-CNN algorithm based on deep learning was significantly improved, and the Dice value, sensitivity, and specificity index data were significantly better than those of the traditional CNN algorithm (*P* < 0.05). In addition, the diagnostic accuracy of MRI images processed by this algorithm was 93.78 ± 5.32%, which was significantly better than the diagnostic accuracy of 64.25 ± 10.24% of traditional MRI images in the control group (*P* < 0.05). In summary, the 3D-CNN algorithm based on deep learning can significantly improve the accuracy and sensitivity of MRI image recognition and segmentation in patients with DME, can significantly improve the diagnostic accuracy of MRI in patients with DME, and has a good clinical application value.

## 1. Introduction

Diabetic retinopathy is currently one of the four leading causes of blindness in western developed countries. In recent years, the incidence of diabetic retinopathy in China has also gradually increased, seriously affecting the visual function and quality of life of diabetic patients [1]. According to statistics, 7% of patients with a history of diabetes for 10 years have retinopathy and about 25% for 15 years. In patients with type 2 diabetes for 20 years, the incidence of oral hypoglycemic agents is 60% and 84% for insulin injection. Diabetes mellitus is a multisystem disease dominated by glucose metabolism disorders, which easily lead to metabolic disorders in retinal tissue, resulting in abnormal retinal vascular function and structure [2, 3]. Diabetic macular edema (DME), defined as a retinal thickening or hard exudate deposition due to diabetes-induced extracellular fluid accumulation within the diameter of one optic disc of the fovea, is one of the common causes of visual impairment in diabetic patients [4, 5]. The Early Treatment Diabetic Retinopathy Study (ETDRS) believes that clinically significant DME (CSDME) needs to meet more than one of the following three conditions: (1) retinal edema thickening is in the 500 *μ*m area from the center of the macula, or less than 500 *μ*m; (2) hard exudation is located in the 500 *μ*m area from the center of the macula, or less than 500 *μ*m, accompanied by adjacent retinal thickening; (3) retinal thickening has at least one optic disc diameter (DD) range, and lesions at any site are within 1 DD from the center of the macula [6].

Current studies suggest that the factors affecting DME formation are as follows: severity of diabetic retinopathy, duration of diabetes, posterior vitreous detachment, pregnancy and cataract surgery, and metabolic control. The pathogenesis of DME may include disruption of the blood-retinal barrier, changes in periretinal vascular dynamics, and periretinal vascular hypoperfusion due to retinal macular ischemia [7, 8]. FFA is a seminal examination that can identify the extent of macular edema, identify the presence or absence of perfusion areas in macular capillaries or capillary rumen-like bulging, and locate the location of leakage points but cannot objectively quantify reactive retinal thickness changes [9]. OCT is currently recognized as the best method to identify the location and severity of retinal edema, and its sensitivity and specificity for detecting macular edema are about 90%, while due to the high cost of MRI examination, the diagnostic accuracy is not comparable to that of OCT, which makes the clinical use of MRI for the diagnosis of DME disease still relatively less [10].

In recent years, various deep learning algorithms have been fully applied in the field of medical image processing, and various image quality optimization algorithms, image fusion processing algorithms, and image segmentation algorithms have emerged endlessly, especially in the field of MRI image optimization [11]. As one of the most widely used and fastest iterative image processing algorithms, the deep learning algorithm is fully applied in the clinical diagnosis of various diseases such as brain tumors, liver cancer, lung cancer, and cervical cancer [12, 13]. However, there are still few reports on deep learning algorithms in the field of DME image processing. Based on this, this study hopes to design a targeted algorithm according to the characteristic information of MRI images of DME patients to realize the rapid segmentation of color vessel regions in the images and the accurate localization and extraction of the characteristic information of injury location.

In summary, this study used the MRI images of the 3D convolutional neural network (3D-CNN) algorithm based on deep learning to monitor DME and comprehensively evaluate the application value of this algorithm by detecting the MRI image quality and diagnostic accuracy of patients with DME. It hopes to provide some reference for the optimization of clinical MRI image diagnosis in patients with DME.

## 2. Materials and Methods

### 2.1. Research Objects and Groups

Ninety-six patients with DME who received treatment in hospital from March 2018 to March 2021 were selected as the subjects, including 54 male patients and 42 female patients, with the age range of 46–73 years, and the mean age was 59.1 ± 13.4 years. These 96 patients with DME included 34 patients with serous retinal detachment (SRD), 29 patients with diffuse retinal thickening (DRT), 17 patients with cystoid macular edema (CME), and 16 patients with mixed DME with coexistence of two or three types. All patients were diagnosed by OCT after admission. They were divided into control group (routinely monitored by MRI images) and experimental group (monitored by MRI images based on deep learning 3D convolutional neural network (CNN) algorithm) according to different treatment methods, with 48 cases for each group. This study has been approved by the ethics committee of hospital, and all subjects included in the study signed the informed consent form.

Inclusion criteria are as follows: (1) patients meeting the clinical diagnostic criteria of DME; (2) detailed records of FFA, OCT, and FP. Exclusion criteria were as follows: (1) macular edema caused by other causes; (2) severe ophthalmic diseases, such as senile macular degeneration, retinal vein occlusion, anterior ischemic optic neuropathy, retinal capillary dilation, retinal vasculitis, and retinal artery occlusion.

### 2.2. Sequence Parameters of MRI Image Diagnostic Examination for DME

The conventional MRI scans of the orbit for DME disease used in this study were located axially and sagittally, including transverse T1-weighted image (T1WI), transverse and sagittal T2-weighted image (T2WI), and transverse fluid-attenuated inversion recovery (FLAR). The scanning parameters of the setup in this study are as follows (Table 1).

### 2.3. Establishment of Segmentation Model of DME Lesions Based on 3D-CNN Algorithm Based on Deep Learning

In this study, the 3D-CNN algorithm based on deep learning is designed to segment DME lesions based on the traditional CNN. This model introduces the time dimension into the convolutional kernel operation. In addition, based on preserving the characteristic information of input data, three-dimensional data are added so that the output data after multiple operations are still arranged according to three-dimensional space.

In the model design, the basic framework architecture of the algorithm is carried out according to the CNN structure. The feature extractor is deployed in the overall structure of the network to make the input data enter the network, and the features of different levels of the image are gradually extracted through the convolution layer, pooling layer, and nonlinear function activation layer. The nonlinear activation function-ReLU function is introduced between the upper and lower input of the activation layer, and then the MRI image segmentation task of DME patients in this study is divided into different output categories. The Dice loss function is introduced to solve the problem of uneven classification in the data processing so as to better realize the MRI image segmentation of DME. Dice score coefficient (DSC) is one of the evaluation indexes based on pixel overlap [14]. The calculation equation of the convolution layer is shown in (1), the mathematical expression of the ReLU function is shown in (2), and the calculation equation of DSC is shown in (3).

(1) $$\begin{array}{c} K=k\left(K_{a-1}\right)*D_{a}+B_{a}. \end{array}$$

K_*a*−1_ represents the feature map information of the previous layer, *B*_*a*_ represents the offset, and *D*_*a*_ represents the weight matrix. Each row in the matrix corresponds to the weight of the neuron connected to all the neurons in the previous layer.

(2) $$\begin{array}{c} \mathrm{ReLU}=\max\left(0,x\right), \end{array}$$

(3) $$\begin{array}{c} D=\frac{2\sum_{j}^{M}g_{j}h_{j}}{\sum_{j}^{M}g_{j}^{2}+\sum_{j}^{M}h_{j}^{2}}. \end{array}$$

In (3), *g*_*j*_ represents the pixel value of point *j* in the prediction results and *h*_*j*_ represents the pixel value of point *j* in the true value label. The sum of *M* pixels is calculated. The gradient equation is shown in (4). The loss function of DME image segmentation can be expressed as in (5).

(4) $$\begin{array}{c} \frac{\partial D}{\partial g_{i}}=2\left[\frac{h_{i}\left(\sum_{j}^{M}g_{j}^{2}+\sum_{j}^{M}h_{j}^{2}\right)-2g_{i}\left(\sum_{j}^{M}g_{j}h_{j}\right)}{\sum_{j}^{M}g_{j}^{2}+\sum_{j}^{M}h_{j}^{2}}\right], \end{array}$$

(5) $$\begin{array}{c} N=\frac{2}{|V|}\sum v\in V\frac{\left(\sum_{j}g_{j,f}h_{j,v}\right)}{\sum_{j}g_{j}+\sum_{j}h_{j}}. \end{array}$$

The number of pixels is represented by *i* in (4), *V* represents a category in (5), and *v* represents a category in the study of the DME segmentation task. *g* represents the segmentation result area predicted by the network, *h* represents the segmentation area calibrated by the true value label, *j* represents any pixel in the image, and *g*_*j*,*f*_*h*_*j*,*f*_ represents the numerical parameters of the predicted output and the output pixel of the true value label area at *j*.

In order to adapt to the dense connection operation in 3D CNN, the jump structure is used to connect the feature maps of each layer in this study, and then the feature maps of all layers are connected in series. On this basis, in order to solve the gradient dispersion problem in the data processing of deep convolution neural network, this study introduces the residual structure [15], and the results of the addition operation between the input data and the output data are correlated by a bypass (shortcut structure). Then the deep network model is constructed.

The corresponding expression of the jump structure is shown in (6), and the output expression of the residual unit is shown in (7).

(6) $$\begin{array}{c} S_{1}=C\left(S_{0},S_{1},S_{2}\ldots S_{j-1}\right), \end{array}$$

(7) $$\begin{array}{c} C_{1}=C\left(S_{0}\right)+V\left(S_{1},\omega_{1}\right). \end{array}$$

The *C*(·) function in (6) represents the fitting target in CNN, and *V*(*S*_1_, *ω*_1_) represents the convolution operation in (7), *ω*_1_ represents the weight parameter in the network, and *C*_1_ represents the network output of the first layer. If *H*(·) is an identity map, it is expressed as (8), then the network output *C*_1_ of the residual structure of the first layer can be expressed as in (9).

(8) $$\begin{array}{c} H\left(S_{1}\right)=S_{1}, \end{array}$$

(9) $$\begin{array}{c} Y_{1}=S_{1}+1=S_{1}+F\left(S_{1},\omega_{1}\right). \end{array}$$

The overall output of the residual structure unit is a linear addition between the original data of the input image and the output data after the convolution operation. In this way, the output of the entire network can be represented by an addition algorithm. Assuming that there are a total of *M* residual structure network units in the network, the overall output of the residual network can be expressed by the following:

(10) $$\begin{array}{c} S_{M}=S_{m}+\sum_{j=m}^{M-1}F\left(S_{1},\omega_{1}\right). \end{array}$$

**Figure 1:**
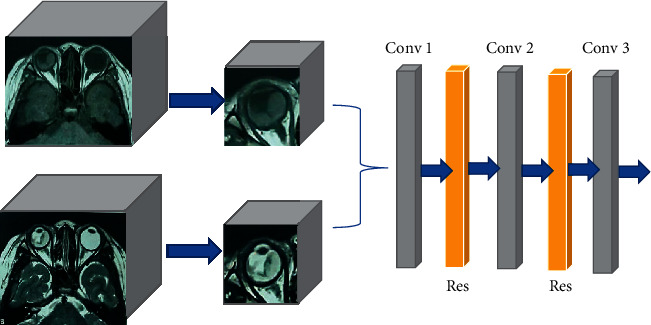
MRI image feature lesion extraction pattern of DME.

**Figure 2:**
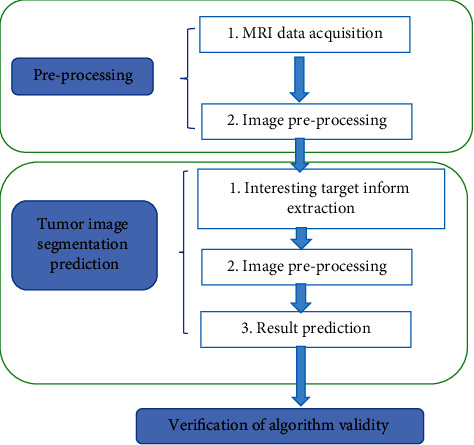
Flow chart of 3D-CNN algorithm based on artificial intelligence.

In this study, the convolution layer setting in the deep learning 3D convolution mode is used to the residual structure unit, and the batch normalization (BN) layer is changed to the group normalization (GN) layer operation in the residual structure. Firstly, the input MRI image information in this model will pass through the convolution layer whose convolution kernel size is set to 3 × 3 × 3, then pass through the GN layer to accelerate the convergence ability of the network, then pass through the nonlinear activation function layer, and will be entered into a convolution layer whose convolution kernel size is set to 3 × 3 × 3, which is used to extract the feature information of the interested target area in the data. The superposition of two 3D convolution layers deepens the depth of the network. Such a structure can greatly reduce the computational complexity without reducing the network performance in the MRI image segmentation task of DME. The structure pattern of the lesion feature extraction network of the DME MRI image is illustrated in Figure 1, and the final algorithm processing flow is illustrated in Figure 2.

### 2.4. MRI Image Quality Assessment Based on Artificial Intelligence 3D-CNN Algorithm

In this study, the experimental environment based on deep learning 3D-CNN algorithm is as follows: 12G Titanx device, Ubuntu16.04 system for related training, and BraTs2017 dataset for algorithm simulation training. The deep learning frameworks Tensorflow and Keras are used.

In this study, the lesion range of the DME area (*S*) outlined by two radiologists was compared with the lesion range determined by the image segmentation of the artificial intelligence algorithm (O). The coincidence degree between the true DME lesion area (Q) and the DME lesion area (O) determined by artificial intelligence algorithm was fitted to calculate the accuracy (Dice), sensitivity, specificity, and Haus distance of the algorithm for MRI imaging diagnosis of patients with DME. The Haus distance can evaluate the degree of fitting between the two different ways to determine the lesion area, which refers to the maximum value of the shortest distance from one point to another point set [16]. The mathematical expressions of Dice, sensitivity, specificity, and Haus distance are shown in (11)–(14).

(11) $$\begin{array}{c} Dice=\frac{|Q\cap O|}{|Q|+\left|O\right|/2}, \end{array}$$

(12) $$\begin{array}{c} \mathrm{Sensitivity}=\frac{|Q\cap O|}{|Q|}, \end{array}$$

(13) $$\begin{array}{c} \mathrm{Specificity}=\frac{|P\cap R|}{|P}. \end{array}$$

*P* and *R* represent other locations outside the real DME lesion area and other locations outside the DME lesion area determined by an artificial intelligence algorithm. In addition, according to the different structural groups of DME, three overlapping tumor regions are divided, which are the whole lesion, the lesion core area, and the enhanced lesion area.

(14) $$\begin{array}{c} \mathrm{Haus}=\max\left\{H\left(Q,O\right),H\left(O,Q\right)\right\}. \end{array}$$

### 2.5. MRI Diagnosis Analysis of DME

In this study, the diagnostic results of MRI images and actual pathological results of patients with DME between the traditional CNN algorithm and artificial intelligence 3D-CNN algorithm were compared, the diagnostic accuracy of patients with DME before treatment and after treatment with the two algorithms was calculated, and the application value of 3D-CNN algorithm based on deep learning for MRI image diagnosis of patients with DME was comprehensively evaluated.

### 2.6. Statistical Methods

The test data were processed by SPSS 19.0 statistical software. The measurement data were expressed as mean ± standard deviation ($\bar{x}$ ± *s*). The comparison of mean between groups was performed by *t*-test. The enumeration data were expressed as percentage (%).. The *χ*^2^ test was used. The differences were statistically significant when *P* < 0.05.

## 3. Results

### 3.1. Summary of Basic Information of Patients in the Two Groups

**Figure 3:**
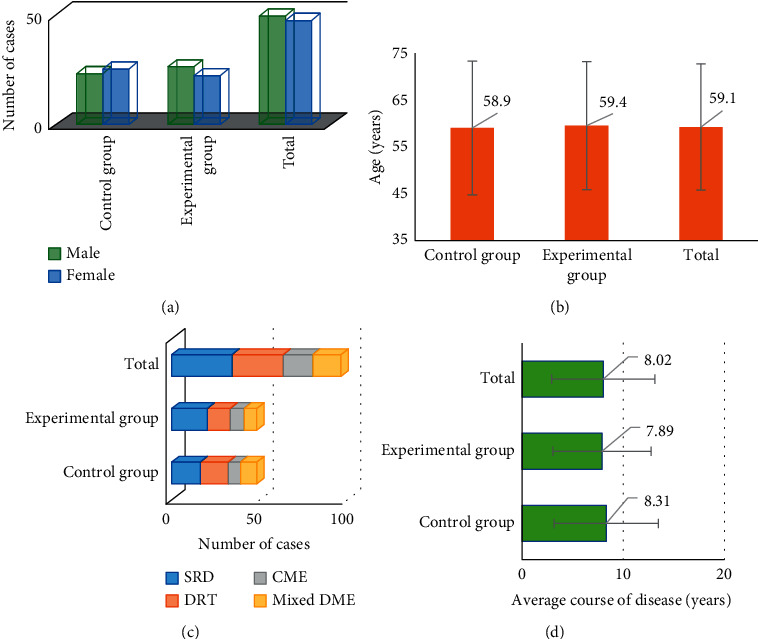
Comparison of basic information between the two groups. (Note: (a) is the gender distribution comparison figure of the two groups, (b) is the mean age comparison figure of the two groups, (c) is the DME type comparison figure of the two groups, and (d) is the mean disease course comparison figure of the two groups).

Figure 3 shows the comparison of basic information of two groups of patients with DME. There was no significant difference in gender distribution, mean age, type of DME, and mean duration of disease between the two groups of DME patients, with no statistical significance (*P* > 0.05).

### 3.2. MRI Images of Patients with DME Processed by Different Algorithms

**Figure 4:**
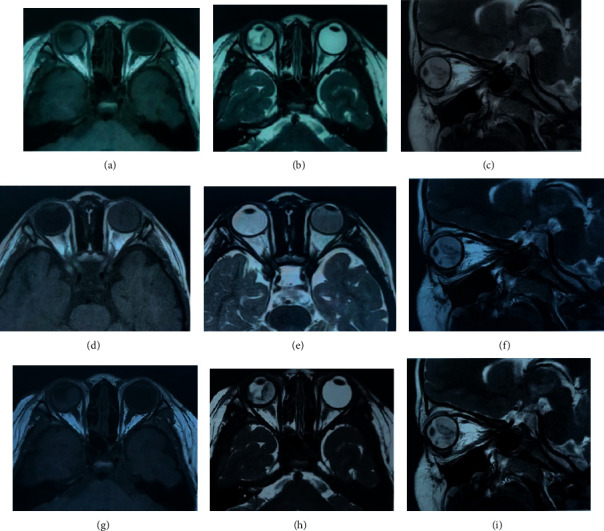
MRI image of patients in the two groups.

**Figure 5:**
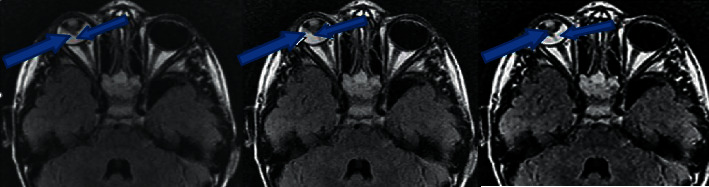
MRI images of patients with DME processed by different algorithms. (a) is sagittal T2WI image of conventional head MRI of a patient in the experimental group, (b) is sagittal T2WI image of head MRI of the patient processed by CNN algorithm, and (c) is sagittal T2WI image of head MRI of the patient processed by deep learning 3D-CNN algorithm. The blue arrow points to the lesion location for the patient.

Figure 4 shows the MRI images of patients in the two groups, and Figure 5 shows the comparison of MRI images of patients with DME processed by different algorithms. Figures 4(a)–4(c) are the MRI images of the experimental group of patients (No. 32), which are the transverse T1WI, transverse T2WI, and oblique sagittal T2WI images of the patient. Figures 4(d)–4(i) are the MRI images of the control group of patients (No. 77) (processed by the deep learning 3D-CNN algorithm). Figures 4(d)–4(f) are the initial MRI images of the transverse T1WI, transverse T2WI, and oblique sagittal T2WI of the patient (No. 77). Figures 4(g)–4(i) are the transverse T1WI, transverse T2WI, and oblique sagittal T2WI MRI images of the patient (No. 77) after processing by the deep learning 3D-CNN algorithm. In the initial state, there was no significant difference in the MRI image quality between the experimental group and the control group. After processing by the deep learning 3D-CNN algorithm, the overall clarity and contrast of the MRI images of the experimental group were significantly improved, the recognition performance of the lesions in the MRI images of patients with DME was greatly increased, and the accuracy of the edge division of the lesion site was significantly improved, making the display of the ocular structure of the transverse and sagittal images of MRI clearer and intuitive, and the difficulty in the diagnosis of macular edema was further reduced.

Figures 5(a)–5(c) are the MRI images of patients from the experimental group, which are the initial MRI images without algorithm processing, the MRI images with conventional CNN algorithm processing, and the MRI images with deep learning 3D-CNN algorithm processing, respectively. Compared with Figure 5(a), the clarity of MRI images in Figures 5(b) and 5(c) is improved to some extent, and the MRI image quality in Figure 5(c) is relatively high. In addition, Figures 5(b) and 5(c) compare stereoscopic and intuitive for imaging the ocular structures of patients with DME, which is more helpful for disease diagnosis.

### 3.3. Evaluation of MRI Image Quality Based on Artificial Intelligence 3D Convolution Neural Network Algorithm Processing

**Figure 6:**
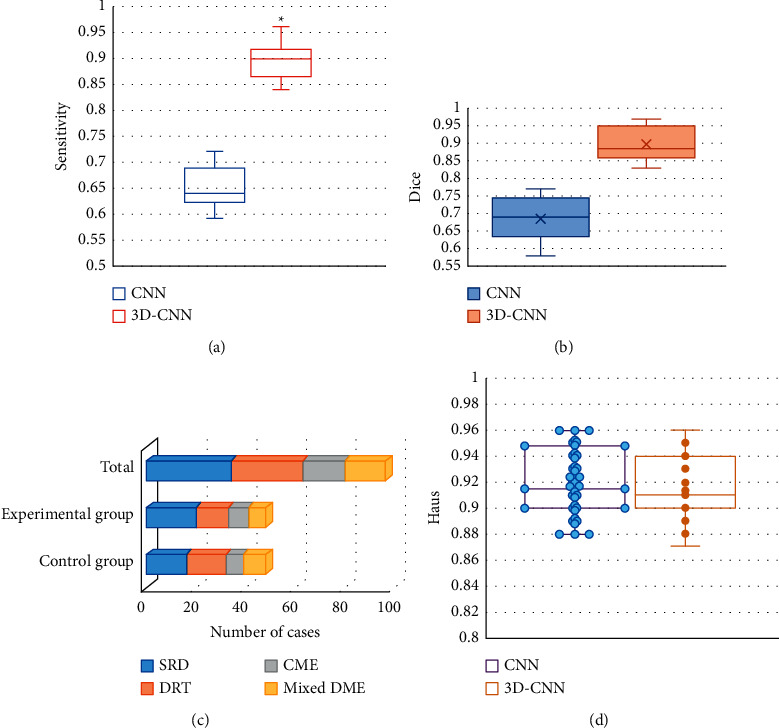
Comparison of image processing quality evaluation indicators of different algorithms. (Note: (a) was the Dice value comparison graph; (b) was the sensitivity value comparison graph; (c) was the specificity value comparison graph; (d) was the Haus distance comparison graph; ^*∗*^ indicated that the difference was statistically significant).

Figure 6 shows a comparison of the diagnostic indicators for the conventional CNN algorithm and the MRI image processing based on the deep learning 3D-CNN algorithm. Figures 6(a)–6(d) are the comparison plots of Dice value, sensitivity value, specificity value, and Haus distance of the two algorithms. The mean Dice value of the MRI images processed by the traditional CNN algorithm is 0.6855, and the resulting interval is (0.58, 0.77). The mean Dice value of the MRI images processed by the deep learning 3D-CNN algorithm is 0.898, and the resulting interval is (0.83, 0.97), with a significant difference between the two groups (*P* < 0.05). The mean sensitivity of the traditional CNN algorithm is 0.64, the resulting interval is (0.59, 0.72); the mean specificity is 0.67, and the resulting interval is (0.61, 0.74); the mean sensitivity of the deep learning 3D-CNN algorithm is 0.9, the resulting interval is (0.84, 0.96); the mean specificity is 0.8855, and the resulting interval is (0.84, 0.93), with significant difference between the specificity and sensitivity of two algorithms (*P* < 0.05). The mean values of Haus distance of traditional CNN algorithm and deep learning 3D-CNN algorithm are 0.920 and 0.916, respectively, and there is no significant difference.

### 3.4. MRI Image Diagnostic Accuracy Evaluation

**Figure 7:**
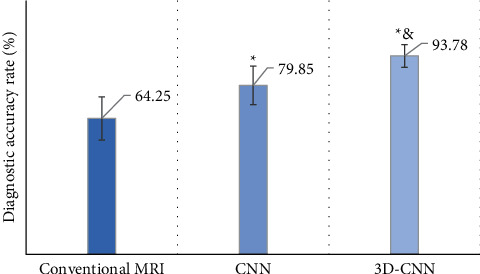
Comparison of imaging diagnostic accuracy of different algorithms. Note: ^*∗*^ indicated that the difference in diagnostic accuracy compared with conventional MRI images was significant (*P* < 0.05); & indicated that the difference in diagnostic accuracy compared with CNN algorithm was significant (*P* < 0.05).

Figure 7 shows the comparison of diagnostic accuracy of MRI images of DME patients with different algorithms. The diagnostic accuracy of conventional MRI images was 64.25 ± 10.24% in the control group and 79.85 ± 9.11% in the experimental group under the traditional CNN algorithm. However, the diagnostic accuracy of MRI images of DME patients in the experimental group processed by the 3D-CNN algorithm based on deep learning was 93.78 ± 5.32%. Compared with the diagnostic accuracy of conventional multimodal MRI images, the diagnostic accuracy of the two algorithms increased significantly (*P* < 0.05), and the diagnostic accuracy of the artificial intelligence 3D-CNN algorithm also increased significantly compared with the traditional CNN algorithm (*P* < 0.05).

## 4. Discussion

In recent years, in-depth learning technology has been continuously updated and iterated and is used as an auxiliary diagnosis and treatment method in the imaging diagnosis process of various common clinical diseases. CNN algorithm based on deep learning has gradually replaced supervised learning as an effective computer-assisted artificial intelligence medical image processing method by virtue of its model learning ability and highly automated target feature information grasping ability [17,] is widely used in the field of medical image segmentation for various diseases such as brain tumors, lung cancer, liver cancer, breast cancer, and gastric cancer, and has achieved good experimental results [18]. At present, the main object of the artificial intelligence imaging algorithm for DME is OCT. Recently, Singh and Gorantla [19] mentioned that the CNN algorithm has a good effect on color fundus image screening in patients with DME.

In this study, a 3D-CNN algorithm based on deep learning was designed according to the MRI image characteristics of patients with DME and used in the clinical MRI image diagnosis of DME disease. The results showed that compared with the MRI images of the control group, the overall clarity and contrast of the MRI images of the experimental group treated with deep learning 3D-CNN algorithm were significantly improved, the recognition performance of the MRI image lesions and the accuracy of the edge division of the lesion site were significantly improved, and the ocular structure display of the transverse and sagittal images of the MRI images was more stereoscopic and intuitive. In addition, compared with the traditional CNN algorithm, the clarity of MRI images and the recognition performance of lesions of the 3D-CNN algorithm based on deep learning were significantly improved, and the Dice value, sensitivity, and specificity index data were significantly better than those of the traditional CNN algorithm (*P* < 0.05). The Haus distance data of the two algorithms were 0.920 and 0.916, respectively, and the difference was not statistically significant (*P* > 0.05). By comparing the clinical diagnostic accuracy of patients with DME treated with different processing methods, the diagnostic accuracy of MRI images processed with the 3D-CNN algorithm designed in this study based on deep learning was 93.78 ± 5.32%, which was significantly better than the diagnostic accuracy of traditional MRI images in the control group (*P* < 0.05) and also significantly improved compared with the diagnostic efficacy of MRI images under the optimization of traditional CNN algorithm (*P* < 0.05). The results are consistent with the study conclusions of Zhang et al. [20], indicating that the MRI image algorithm based on deep learning can improve the MRI image quality to a great extent, optimize the identification process of disease characteristics images, and improve the efficiency of disease diagnosis. However, the relevant optimization procedure of the 3D-CNN algorithm based on deep learning is very complex and remains to be further studied.

It shows that the deep learning algorithm has a good utilization value in MRI image monitoring of DME and can well provide some expansion ideas for improving the clinical diagnostic accuracy of patients with DME from the imaging point of view.

## 5. Conclusion

A deep learning 3D-CNN algorithm for MRI image characteristics of DME patients is designed and applied to the clinical diagnosis of DME patients. The results showed that the intelligent recognition and segmentation performance of the 3D-CNN algorithm based on deep learning for MRI images in patients with DME was significantly improved compared with the traditional CNN algorithm, and the diagnostic accuracy was also significantly improved.

However, there are some shortcomings as follows: the sample size of patients with various types of DME is relatively small and the corresponding algorithm optimization analysis is not performed for the MRI image characteristics of patients with different types of DME. In addition, the monitoring index of MRI image segmentation quality based on the deep learning 3D-CNN algorithm used in this study was relatively small, and the performance evaluation of this algorithm was relatively single. Then, the algorithm will be further optimized, and more performance evaluation indexes will be included to perform multiangle comprehensive analysis for the application value of this algorithm. In conclusion, this study confirmed that the image characteristics of MRI based on deep learning algorithm have a good application value in the clinical diagnosis of DME patients, which is worthy of further clinical promotion and provides a reference basis for the imaging diagnosis, treatment, and monitoring of clinical DME patients.

## Figures and Tables

**Table 1:** MRI scanning parameters of DME.

	MRI scanning position			
	T1WI transverse position	T2WI sagittal position	T2WI transverse position	FLAR transverse position
Number of collections	Single	Single	Single	Single
Time of repetition (TR)	250 ms	440 ms	4000 ms	6000 ms
Time of echo (TE)	2.46 ms	2.46 ms	93 ms	96 ms
Flip angle (FA)	70°	90°	120°	130°
Matrix	320 × 320	320 × 320	320 × 320	256 × 256
Layer thickness	6 mm	4 mm	6.5 mm	6 mm
Layer spacing	0 mm	0 mm	0 mm	0 mm
Field of view (FOV)	320 mm × 320 mm	320 mm × 320 mm	240 mm × 240 mm	320 mm × 320 mm

## Data Availability

The data used to support the findings of this study are available from the corresponding author upon request.
